# National Necessities and Sacrifices

**Published:** 1918-04-13

**Authors:** 


### The Hospital

The Workers' Newspaper of Administrative Medicine and Institutional Life, Administration, National Insurance and Health.

No. 1662, Vol. LXIV. SATURDAY, APRIL 13, 1918. PRICE ONE PENNY

### NATIONAL NECESSITIES AND SACRIFICES.

Whatever other conclusions issue from the recent fighting on the Western Front, it is certain that the event imposes fresh sacrifices on the nation. The one consolation about these sacrifices is that their necessity is clear beyond dispute. There is no alternative, or, at least, the only alternative is one to which our people will not listen for a moment. When, as we have been told on high authority, it is a question of "going on" or "going under" there is no difficulty about the reply, and still more when, as here, the issue is presented not in words only, but in happenings evident to all, the decision is taken without delay. Necessity is a great help to vigorous action, for it leaves no opportunity for the hesitations and debates which proverbially hinder enterprise. When the path is clearly indicated the whole sum of available effort may be devoted to its pursuit. This is the position in which the nation finds itself to-day, and there is stimulus in its recognition. Some of the sacrifices contemplated are indeed grievous, and they add to a catalogue of loss and suffering pathetic beyond words. But can they be avoided? To such a question there is no affirmative reply compatible with either national honour or national safety, so that even here hesitation has no opportunity. We would all, indeed, fain see some chance to spare the manhood of our people from the perils and horrors of further war. But no such chance exists, unless we are content to cease the struggle for human freedom and opportunity. To do this we most certainly are not content, and hence we are shut up to the sacrifices which are involved in our resolution. This is the sharp issue which is presented in the first place to those who stand charged with the national interests, and from these the decision comes down to the body of the people. Both to one and the other this element of necessity and obligation carries an element of clear vision and mental resolution. What was seen without confusion or alloy in the early days of the war is seen again in this latest stress. There is no escape from the bond of duty, and hence the hard resolve and its consequences are accepted with calmness and determination. There may be differences of opinion about the methods and arrangements to be pursued, but there is no debate as to the necessity. The goal has to be reached whatever the suffering, and from this point of departure all else follows. Those who cannot fight must serve, and the individual who is neither in one category nor the other will have difficulty in justifying his existence.

We are quite sure that in this national resolve our medical readers take their full share. The record of the profession in relation to the war needs words neither of compliment nor of flattery. It contains a story of much fine service and much individual sacrifice. There is now a call for further service and sacrifice, and these, we doubt not, will be promptly forthcoming. What has occurred in recent weeks has consequences which can be appreciated quite apart from any official declarations. Fighting such as has been the lot of our armies in France for a fortnight or more inevitably means a terrible toll of injured and wounded men, and these men, all will agree, must have medical succour and care. Again, a retiral of the fighting line before an overwhelming rush of the enemy leaves medical officers engaged in their humane tasks exposed to almost inevitable capture. There is thus a double reason for the claim received from the Army for still more help from the medical profession. It is, indeed, a severe claim to ask men who have already given up their practices to serve in the R.A.M.C. and who after such service have returned to civilian work—it is, admittedly, a severe demand to ask these men once more to come back to military duties. But what is the alternative? It is, we fear, that our wounded and stricken soldiers will get something less than adequate help, and such a position none can contemplate without shrinking. The official appeal on this point recognises the sacrifice which is asked, and, indeed, the sacrifice cannot be minimised. But it is avoidable only at a price which no lover of his country and his country's cause is willing to pay, and hence it must be accepted as a high duty and obligation. It has necessity in relation to an approved and great cause, and here is the strength of its demand.

It is not only the younger members of the profession on whom the pressure of further sacrifice is to fall. With the raising of the age standard of military service there will be included as liable to conscription a number of doctors who have previously been outside this area of compulsion. Many of these men are already giving voluntary service in some form or other, and even in a great crisis the medical needs of the civilian population cannot be altogether ignored. Indeed, it is practically certain that in some parts of the country the number of civilian practitioners has been reduced to a point below rather than above the safety line. There are, however, other districts, residential for the most part, where some economy may even yet be practised, and, in view of the existing emergency, service will certainly be asked. Of course, it is a serious matter to take middle-aged men from their homes and habits and to transplant them into a new atmosphere and to new duties. But here, as before, we ask, What is the alternative? If the nation—and the medical profession certainly not less than their fellow-citizens—is resolved, as it is resolved, to see this thing through whatever the cost, no one can avoid the claim which presses individual interests and convenience into the second place and demands the sacrifices necessary to the achievement of the national purpose.

